# Cryoablation of cardiophrenic angle lymph node metastases: a case report

**DOI:** 10.1186/s13256-017-1313-4

**Published:** 2017-08-14

**Authors:** Xiaomei Luo, Weibing He, Xinan Long, Gang Fang, Zhonghai Li, Rongrong Li, Kecheng Xu, Lizhi Niu

**Affiliations:** 1 Medical College, Jinan University, Guangzhou, 510632 Guangdong Province China; 2 Department of Oncology, Fuda Cancer Hospital, Jinan University School of Medicine (Guangzhou Fuda Cancer Hospital), Guangzhou, 510665 China; 3 Department of Surgery and Anesthesia, Fuda Cancer Hospital, Jinan University School of Medicine (Guangzhou Fuda Cancer Hospital), Guangzhou, 510665 China; 4 Department of Radiology, Fuda Cancer Hospital, Jinan University School of Medicine (Guangzhou Fuda Cancer Hospital), Guangzhou, 510665 China; 5 Department of Ultrasound, Fuda Cancer Hospital, Jinan University School of Medicine (Guangzhou Fuda Cancer Hospital), Guangzhou, 510665 China; 6 Guangzhou Fuda Cancer Hospital, School of Medicine, Jinan University, No. 2, Tangdexi Road, Tianhe District, Guangzhou, 510665 Guangdong Province China

**Keywords:** Cryoablation, Lymphatic metastasis, Cardiophrenic angle lymph node

## Abstract

**Background:**

Cardiophrenic angle lymph node metastases are relatively rare. Surgical resection is the main treatment for cardiophrenic angle lymph node metastasis, but it is not always possible.

**Case presentation:**

Here, we report our initial experience with cryoablation of a cardiophrenic angle lymph node metastasis from liver cancer. As the cardiophrenic angle lymph node metastasis was located close to the heart, about 200 mL of 0.9% saline was injected into the pericardium to separate the heart from the target area. The cardiophrenic angle lymph node metastasis was successfully ablated, without any complications.

**Conclusions:**

Cryoablation may be a suitable alternative treatment for cardiophrenic angle lymph node metastasis.

## Background

Cardiophrenic angle lymph nodes (CPLN), which are located behind the xiphoid process in the gap between the diaphragm and the heart, are an unusual site for metastases [1]. Although cryoablation has been explored as an option for treating metastatic lesions in patients who cannot undergo surgery or chemotherapy [2], there are no reports of cryoablation techniques applied to CPLN metastasis. Here, we describe a patient with CPLN metastasis from a liver cancer who was successfully treated with cryoablation. The Institutional Review Board of our hospital approved this report.

## Case presentation

**Fig. 1:**
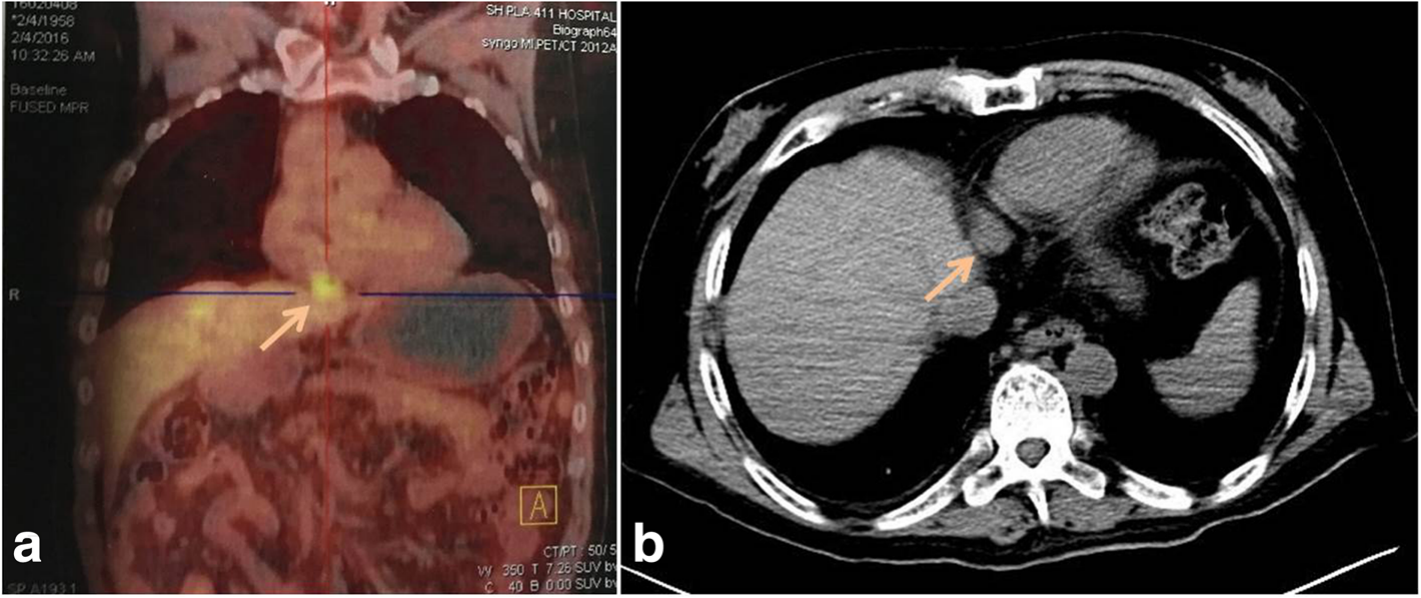
Preoperative images. **a** Follow-up positron emission tomography/computed tomography image at the local hospital; the *arrow* indicate the cardiophrenic angle lymph node metastasis. **b** Computed tomography image at our hospital; the size of the node is about 2.3 × 1.7 × 1.8 cm (*arrow*)

The patient, a 57-year-old Asian man, was referred to our hospital in February 2016 for treatment of CPLN metastases from liver cancer. In July 2011 he had undergone liver transplantation at a local hospital and had been on antirejection drugs since then. In 2013, at a follow-up review, his serum alpha fetoprotein (AFP) level was seen to be persistently elevated, and the patient was treated with oral sorafenib targeted therapy for 1 year. However, the response was poor, and multiple targeted drugs had been administered since then. In February 2016, a follow-up magnetic resonance imaging (MRI) scan showed an enlarged right cardiophrenic lymph node. A positron emission tomography/computed tomography (PET/CT) examination showed an active nodule between the heart and the diaphragm suggestive of lymph node metastases, involving the parietal pericardium (Fig. 1a). The patient was then referred to our hospital for further treatment.

A CT examination at our hospital revealed an enlarged, 2.3 × 1.7 × 1.8 cm, right CPLN, consistent with local metastasis (Fig. 1b). A CT-guided percutaneous lymph node biopsy was performed, and histologic examination confirmed metastasis. Our patient was unwilling to undergo surgery and requested minimally invasive treatment. We explained the options, and our patient chose to undergo cryoablation.

**Fig. 2:**
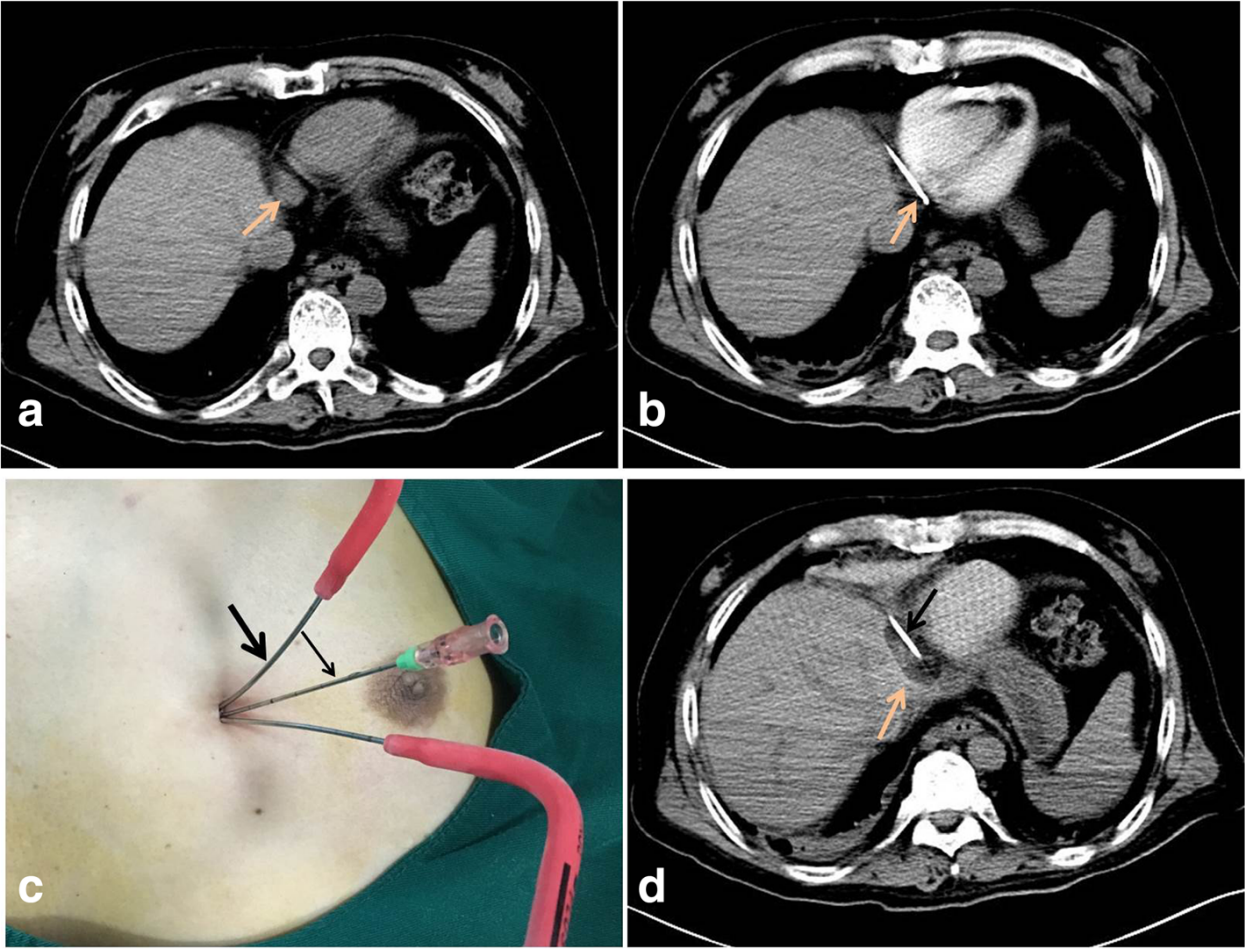
Intraoperative images of the patient. **a** Computed tomography image before cryoablation; the *arrow* indicates the tumor. **b** An 18G needle (*arrow*) was inserted into the pericardium and 200 mL of 0.9% saline solution was injected. **c** The *thick arrow* indicates the cryoprobes and the *thin arrow* indicates the 18G needle. **d** Computed tomography image of the ice ball (*pink arrow*) taken during the procedure to confirm that the ice ball had reached a sufficient size (3.5 × 3 × 2.8 cm^3^). The *pink arrow* indicates the cryoprobe

Our patient was fasted for 6 hours before the procedure. The procedure was carried out under CT guidance (SOMATOM Definition 64 AS; Siemens Medical Solutions, Forchheim, Germany), using an argon and helium gas-based system (Endocare, Irvine, CA, USA), two 1.47-mm cryoprobes (Endocare), and a thermal sensor. With our patient supine, the right side of his chest wall was selected as the puncture site and the right cardiophrenic angle as the target tumor area. The skin at the puncture site was anesthetized with 2% lidocaine 5 mL plus 0.75% bupivacaine 5 mL. An 18-gauge needle was inserted into the pericardial cavity under CT guidance. Subsequently, 10 mL of 0.9% saline solution was injected to identify if the tip of needle had entered into the pericardium. Once this was established, about 200 mL of 0.9% saline was injected so that the tumor was well revealed and a safe puncture path was available (Fig. 2b). Next, with CT guidance and continuous close monitoring of vital signs, two 1.47-mm cryoprobes were carefully inserted into the target node in the right cardiophrenic angle (Fig. 2c). Ablation was performed with two 7-minute freezing cycles, with freezing temperature of −130 °C to −150 °C, followed by 3 minutes of thawing. CT was used to visualize the size of the ice ball during the procedure (Fig. 2d) and to confirm that the ice ball had reached sufficient size (3.5 × 3 × 2.8 cm), with a margin of 5 mm beyond the CPLN metastases. The cryoprobes were removed after rewarming. The saline injected into the pericardial cavity was aspirated, the needle withdrawn, and the puncture point was bandaged. His vital signs remained stable throughout the procedure. The total intraoperative blood loss was 2 mL.

**Fig. 3:**
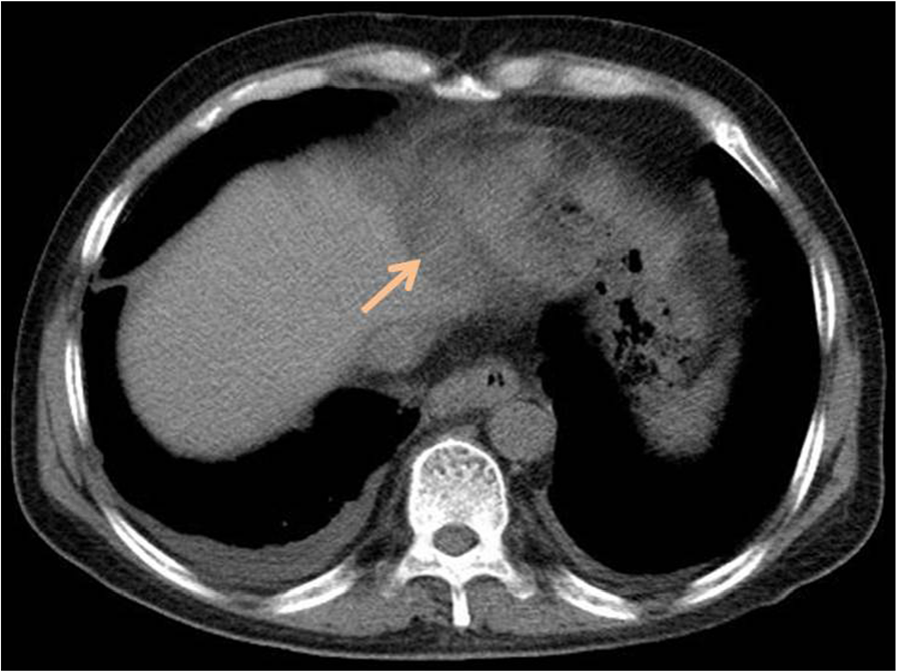
Computed tomography scan images obtained 3 day after cryoablation. The *arrow* indicate the ablated zone

No minor or major complications were noted during or after the procedure. A follow-up CT scan performed 3 days after cryoablation (Fig. 3) showed a 3.5-cm-diameter ablated zone in the right cardiophrenic angle.

**Fig. 4:**
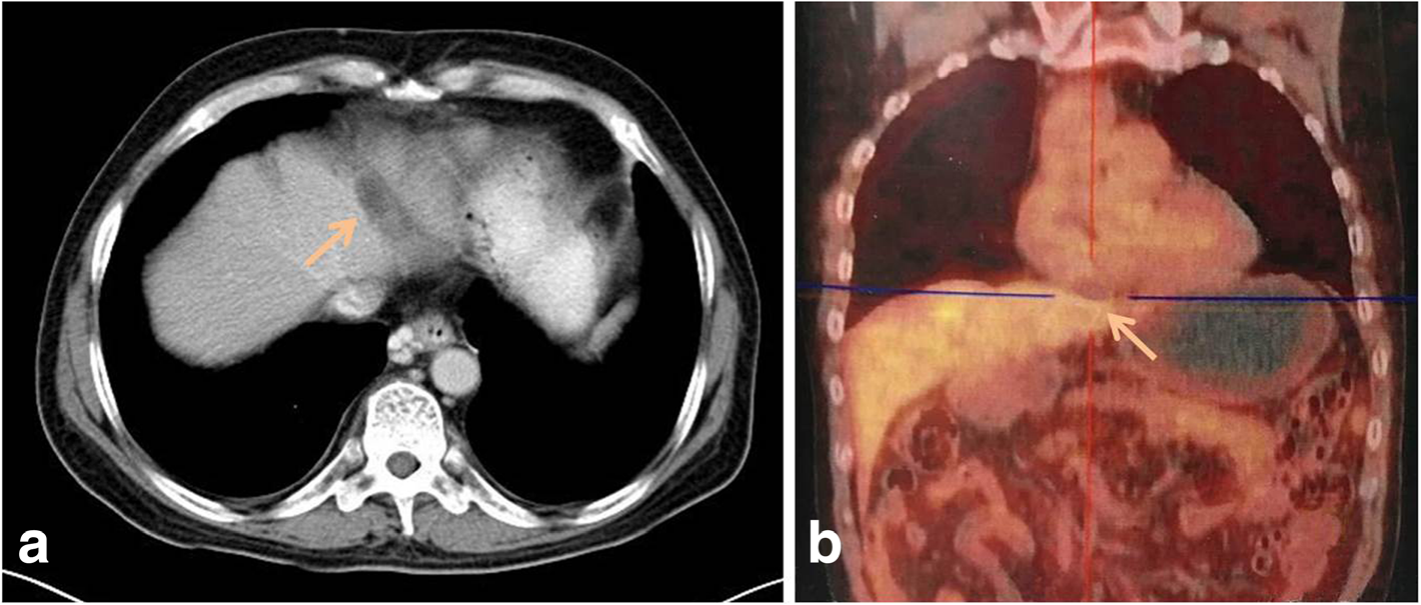
Follow-up imaging examination after cryoablation. **a** Follow-up computed tomography scan 1 month later showed a 3.5 × 1.8 × 1.7 cm ablated zone, with no evidence of recurrence. **b** Follow-up positron emission tomography–computed tomography examination six month later showed no evidence of recurrence. The *arrows* indicate the ablated zone

A follow-up CT scan 1 month later showed a 3.5 × 1.8 × 1.7 cm ablated zone, with no evidence of recurrence (Fig. 4a). Six months later, a follow-up PET/CT examination showed no evidence of recurrence (Fig. 4b).

## Discussion

The presence of lymph node metastases will upstage a tumor, adversely affect prognosis, and influence treatment choice [1]. Isolated CPLN metastases are relatively rare [3]. While CPLN may be seen in normal subjects, they are usually less than two in number and measure less than 5 mm in diameter [1]. The afferent lymphatics to the CPLN arise from the pericardium, anterior thoracoabdominal body wall, pleura, and parts of the diaphragm, which itself drains lymph from the peritoneum [4]. Tumor lymph drainage is usually along well-recognized lymphatic pathways, but unusual lymph node sites can be involved and may sometimes be the only site of disease, particularly in patients with recurrence.

Cryosurgery is a novel therapeutic approach in benign and malignant tumors, and is especially useful in unresectable tumors [5]. Encouraging results have been reported in lung [6], liver [7], prostate [8], renal [9], and breast cancer [10]. Cryoablation is an attractive option for treating unresectable CPLN metastases.

Cryoablation induces tissue damage mainly through two separate freezing-related events: a direct toxic effect on the cells and an indirect effect via actions on the tumor vasculature, with the end result being a coagulative necrosis [11]. The direct effect involves intra- and extracellular ice formation at temperatures below 0 °C and enzymatic and cell membrane dysfunction, resulting in osmosis of water out of the cells and cellular dehydration. During the thawing cycle, water returns to the intracellular space and causes cellular lysis. The indirect effect is the result of occlusion of small blood vessels because of ice formation [11]. The temperature at the margin of the ice ball is 0 °C, a temperature at which tumor cells can survive; however, 5 mm inside the edge of the ice ball, the temperature is approximately −20 °C to −50 °C, at which temperatures tumor cells are killed. Therefore, in order to achieve complete ablation of tumor tissue, the edge of the ice ball must extend 5 mm beyond that of the tumor [12]. Therefore, in our patient, a margin of at least 5 mm of normal tissue was frozen circumferentially around the tumor.

A major limitation of cryoablation is incomplete destruction of cells at the border of the treated regions where the tissue temperature is greater than −20 °C. The irregular shape of the CPLN and infiltration of surrounding organs also give rise to complications during cryotherapy. For these reasons, adjunctive approaches are required and cryotherapy is usually combined with other treatments. Several authors have reported the use of 5% dextrose in water as the artificial fluid [13, 14]. Because friction between ions is responsible for the heating in radiofrequency ablation (RFA), 5% dextrose in water—which is isotonic and nonionic—could be the ideal buffer solution during RFA. Oura *et al*., in their study on RFA of breast tumors, reported few skin burns with the use of 5% dextrose in water as the buffer fluid. The fluid protected against skin burns by increasing the distance between the tumor and the skin and by interrupting the radiofrequency waves [15]. Since the heating during microwave ablation is the result of vibration of dipolar molecules rather than the friction of ions, Zhang *et al*. chose 0.9% saline to create an artificial pleural effusion during microwave ablation of liver tumor and achieved good results, with no complications [16]. In our patient, the CPLN metastasis was located close to the heart, and we chose 0.9% saline solution to create an artificial pericardial effusion to separate the tumor from the heart during cryoablation. This seems to be a feasible approach as our patient tolerated the procedure without any complications.

## Conclusions

In conclusion, our results show the potential of cryoablation as a new treatment method for CPLN, particularly in patients in whom surgery is contraindicated. Cryoablation may be a suitable adjunctive therapeutic option for pure CPLN metastasis. However, further prospective investigations with long-term follow-up are needed to confirm our findings.

## Abbreviations

AFP: Alpha fetoprotein
CPLN: Cardiophrenic angle lymph nodes
PET/CT: Positron emission tomography/computed tomography
RFA: Radiofrequency ablation
